# Prevalence of Human Hydatidosis Based on Hospital Records in Hamadan West of Iran from 2006 to 2013

**Published:** 2017

**Authors:** Nazanin FALLAH, Khadijeh RAHMATI, Mohammad FALLAH

**Affiliations:** 1. Dept. of Anesthesiology, School of Medicine, Besat Hospital, Hamadan University of Medical Sciences, Hamadan, Iran; 2. Dept. of Parasitology and Mycology, School of Medicine, Hamadan University of Medical Sciences, Hamadan, Iran

**Keywords:** Human, Hydatidosis, Surgery, Hospital records, Iran

## Abstract

**Background:**

The reservoir and intermediate hosts of *Echinococcus granulsus* and human hydatidosis are more prevalent in the Alborz and Zagros Mountains slop due to rich pastures for sheep raising. Many cases of hydatid cysts operate in local hospitals annually. The present study aimed to review the epidemiologic characteristics of the hydatidosis patients.

**Methods:**

In this descriptive study, the medical files of 182 hydatidosis patients from main public and private hospitals in Hamadan Province, western Iran from 2006 to 2013 were reviewed. The data collected from eight general hospitals including demographic data, clinical and diagnostic measures, surgical approaches, and outcome were entered into the prepared checklist and analyzed by descriptive statistics.

**Results:**

All patients were diagnosed and operated giving an average of 26 cases per year, or 1.5 cases per 100000 inhabitants. The site of cysts was as follows: liver 70.9%, lung 24.7%, and in both these organs 2.2%. The ratio of male and female patients was approximately 1:1. Mean age of patients was 44.5±21.5 yr at range of 3 to 91 yr. The majority the patients were illiterate (32.2%) and resided in the rural areas (61.7%), and their occupations were housewives (36.8%). Almost 90% of patients diagnosed by imaging methods and 8% had history of surgery for cyst.

**Conclusion:**

Hydatidosis is a major health problem still in this region and more extensive epidemiological investigations of CE is necessary to better determine the prevalence, economic impact and risk factors for the disease control.

## Introduction

Hydatidosis is one of the most important zoonotic parasitic diseases in the world and in Iran as well (1, 2). Hydatidosis or cystic echinococcosis (CE) is the larval cystic stage (or metacestode called hydatid cyst) of a small canid tapeworm (*Echinococcus granulosus*) that may cause illness in intermediate hosts, generally herbivorous animals and humans life cycle of parasite and wide spread range of final and intermediate hosts in the domesticated and wild animals, the control of disease in humans is very difficult (3).

In the normal life cycle of *E. granulosus*, adult tapeworms inhabit the small intestine of carnivorous definitive hosts, such as dogs, fox, or wolves, and larval cyst stages occur in herbivorous intermediate hosts, such as sheep, cattle, camel, and goats. A number of other suitable intermediate hosts, such as pigs, horses and wild herbivores, are involved in the life cycle in many parts of the world (4).

In the most sheep-raising countries where hydatid disease is endemic, such as the Middle East and North Africa, South America and Australia; typical dog-sheep cycle plays main role in epidemiology of parasite (5, 6).

Human infection occurs most frequently when individuals handle or contact infected dogs or other infected carnivores or inadvertently ingests food or drink contaminated with fecal material containing *E. granulosus* eggs.

The surveillance of human hydatidosis that more often perform on hospital records can provide useful information on the disease burden at the community level aside with the epidemiological data regarding livestock and definitive host of parasite, particularly dog, can provide comprehensive information for policy makers of health system. In addition, analyze of epidemiologic factors obtained from such as these studies could clear the main risk factors for human infection in each area.

The first survey on the human hydatidosis cases in the Hamadan was done at the 1980s (7) that report showed the importance of disease in this region. There are various reports of human hydatidosis as case reports or survey on the operated hydatid cyst cases from different provinces of Iran but, such as these reports are rare and few from Hamadan Province (8,9). A report indicated the high frequency of surgery for hydatid cyst in the Iranian hospitals, even up to 1% of all surgeries (10).

The present study aimed to review the epidemiologic characteristics of the hydatidosis patients such as gender, age, literacy, residence; and clinical features of disease such as diagnostic approaches, size, fertility and location of cysts, complications, morbidity and mortality and outcome of treatment of hydatid cysts during the period 2006–2013 in public and private hospitals of Hamadan Province, Iran.

## Materials and Methods

### Surgery wards

Totally, there are 20 hospitals in the Hamadan Province, western Iran. Two hospitals are supervising by Social Security Organization (SSO), one by army, one depends to a Charity Institution (Mahdieh) and one hospital is administered by private sector (Bu-Ali Hospital). The other hospitals are administered by Medical Sciences Universities in the main cities Hamadan, Malayer, and Nahavand. Five hospitals are teaching hospitals and rests are treatment hospitals only. The population of province is about 1700000 according to 2011 census, that 600000 reside in the Hamadan, center of province. All medical records of hospitals that had surgery ward searched for hydatidosis patients.

### Data collection

All medical records on the diagnosed and operated hydatid cyst cases in the hospitals of the Hamadan Province that had surgery ward (including Hamadan City, Malayer, Nahavand hospitals as university hospitals and Atieh Hospital (SSO), Mahdieh and Bu-Ali Hospital); for a 7 yr period from 2006 to 2013 were collected and reviewed. The relevant data extracted in the datasheets prepared prior to the study.

After extracting the data, they entered into the computer and analyzed by SPSS software (Chicago, IL, USA). In general, hydatid cysts had been operated in the 8 general hospitals in the province. The patients’ file was relatively complete in the university teaching hospitals but there was minimum well-documented data about the patients and their history that had surgery in the private hospital. All records and files related to history of patients had been fired, except files of recent three years in the private hospital. Therefore, unfortunately, we could obtain only name, age, and gender of operated patients in addition to site of cysts, for 3 of 7 studied years from private hospital.

## Results

From 2006 to 2013, all confirmed hydatid cyst cases operated in these province hospitals were analyzed. Hospitalization records were reviewed and confirmed cases of hydatid cyst were classified according to ward of residence, age, sex, cyst location and cyst type. A total of 182 hydatidosis patients were diagnosed that giving an average of 26 cases per year, with respect to province population (1700000 inhabitants), equivalent to approximately 1.52 cases per 100000 inhabitants per year.

**Fig.1:**
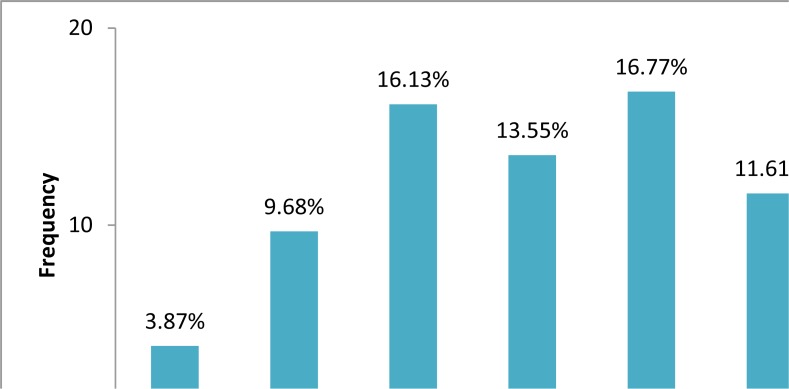
Age distribution of operated hydatid cysts in Hamadan Province, 2006–2013

The site distribution of 182 cysts recorded was as follows: liver 70.9%, lung 24.7%, in both these organs 2.2%; and spleen and ovary less than 1%. The ratio of male and female patients was approximately 1:1 (females 52%, males 48%; *P*>0.05%). Mean age of patients was 44.5±21.5 yr at range of 3 to 91 yr. Most cysts are diagnosed in the patients of the age group 20–50 yr (47.2%) and then in the age groups higher than 51 yr (38%) (Fig. 1).

**Fig.2:**
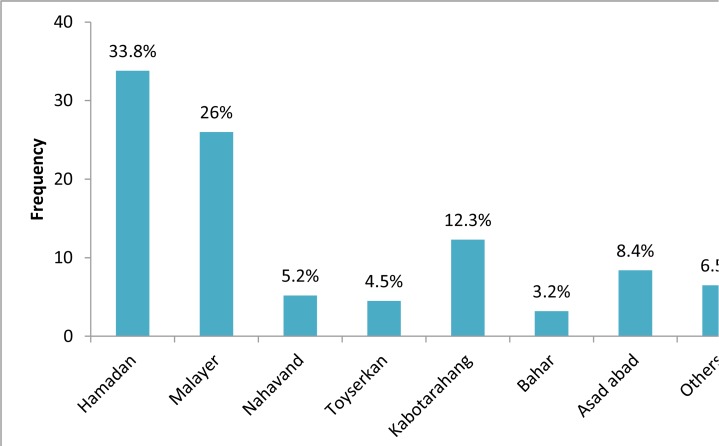
Distribution of hydatidosis patients in different cities in Hamadan Province The main diagnostic methods are showed in Fig. 3.

The cysts in this study were observed in different sizes. At the time of diagnosis, the diameters of the cysts had been determined by imaging techniques and the reported sizes were 10 through 190 mm. Size of majority of cysts (47.1%) was in the range of 50–100 mm. The average cysts diameter was 78.13±40.8 mm. The most cysts were in the 5^th^ decade of the life and most of patients were from Hamadan, and Malayer (Fig. 2).

**Fig. 3:**
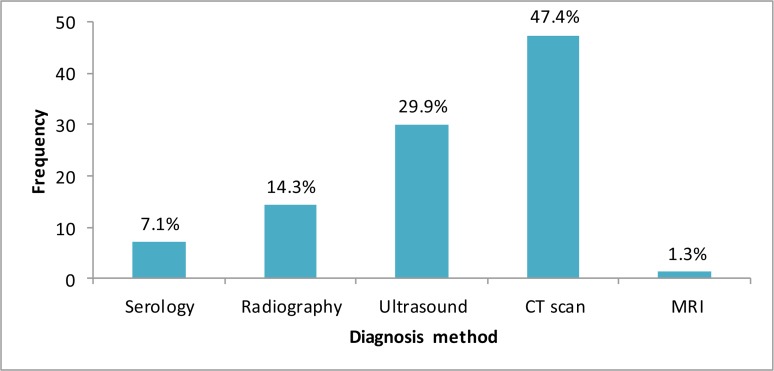
Main diagnosis methods in the patients

The majority of the patients were illiterate (32.2%) and inhabitant in the rural areas (61.7%) and their occupations were housewife (36.8%). Finally, almost 90% of patients were diagnosed by CT scan and other imaging methods, and 8% of them had history of another surgery for cyst. Chief complaints and laboratory findings in the patients are showed in the Table 1 and 2.

**Table 1:** The chief complaint of patients with hydatid disease

***Sign and symptom***	***Patients N(%)***
Abdominal pain	53 (29.1)
RUQ pain	31 (17)
Epigastric pain	23 (12.6)
Nausea and Vomiting	15 (8.2)
Anorexia	9 (4.9)
Fatigue	8 (4.3)
Hepatomegaly	3 (1.6)
Icterus	3 (1.6)

**Table 2:** Laboratory findings of the 182 hydatidosis patients

***Factors***	***Mean and SD***
WBC	3990–23500 (mean: ± 2942)
SGOT	41–322 (mean: 96.6±76.91)
SGPT	42–585 (mean: 122.47±121)
ESR	31–137 (mean: 85±30.95)
Albumin	2–3.4 (mean: 2.83±.62)
Amylase	33–2184 (mean: 211.38±592)
Alkaline phosphatase	3–1069 (mean: 480.3±234.5)
Bilirubin	0.2–8.6 (mean: 2.28±2.56)

## Discussion

This study indicates the incidence of human hydatidosis is about 1.5/100000 of population in this province that is higher than some previous reports, 0.6–1.2/10000 and 0.8/100000 (2, 22). However, the overall prevalence of *E. granulosus* infection in the stray dogs reported 48% in the city of Hamadan previously (11), and prevalence of hydatid cyst in the intermediate hosts reported from 12.3% (2009) through 6.5% (2014) in the sheep and goats in the abattoir of this city (12, 13); the rates that represent hydatidosis is a major public health threat in this province.

Although patients’ age ranged from 3 to 91 yr old but the most patients were in the third, fifth and seventh decade of life, however, the age relationship was not significant statistically. According to most of the studies, peak incidence of the disease was between 20 to 40 yr old (7, 9, 10, 22). Although the incidence of disease was relatively, lower in the ages below 19 yr old it seems all ages are at some risk of infection to hydatid cyst in this province.

The majority of cases from Hamadan, center of province, and Malayer, two major cities of province that concentration of treatment and diagnostic properties in these cities and referring the patients and not due to higher infections of these areas. Liver was the most affected organ (70.9%). Other infected organs included only the brain and spleen, though both liver and lung infection was only in one case.

In contrast to other reports, the sex ratio of infection between male and female in this province was approximately 1:1 that indicate both gender, like different ages, are at the same risk of infection because of the special cultural and social behaviors.

This study showed serological methods less used for diagnosis of HC and because of the accessibility, using portable sonography tools and CT are the main routes of the diagnosis. The physicians prefer the accessible diagnostic tools, such as CT and ultrasound for diagnosis of hydatid cyst because of the simple and quick performance and perhaps lower false positive of negative results (14). The advent of modern imaging techniques, in particular, ultrasound and CT scan, represented not only a breakthrough in the diagnosis, treatment, and follow-up of hydatidosis patients, but also for clinicians have been striving for an imaging based classification of hydatid cysts and to correlate individual cyst stages with the natural history and treatment-induced involution processes of the cysts (15).

The chief complaint in 53 out of 182 cases (29%) was abdominal pain; however, 31 patients (17%) had RUQ pain because of the high incidence of lung involvement. Other symptoms related to digestive systems such as gastric pain, nausea, and vomiting, anorexia and fatigue could be the noticeable and different finding in this study.

These cases report herein could not be the all cases of the hydatidosis in this province because; some patients may refer to cities out of this province, including Tehran seeking the therapeutic measures.

Hamadan Province has a traditional culture and the behavior of people in relation to the animal could facilitate the hydatid cyst infection. It is an increasing public health and socioeconomic concern, in different parts of Iran. It is currently considered an emerging or re-emerging disease and the geographic distribution and its spread are greater than previously believed (16–18). CE is theoretically an eradicable disease, but numerous factors are involved in the maintenance of the cycle in an endemic area, including behavioral and cultural factors that are often difficult to regulate or modify quickly in different communities.

In Iran, the prevalence of CE is relatively high in both human and animal reservoirs, especially in the slops of Alborz and Zagros Mountains and highest rates of transmission to humans are associated with *E. granulosus* in the dog/sheep cycle (19, 20). In most parts of country, livestock production systems include predominantly traditional animal husbandry, characterized by pasturalism, uncontrolled animal movements, and nomadism (19). Unfortunately, home-slaughter practice is still common in the most parts of country. In addition to livestock slaughter out of the abattoirs, there are a high number of festivals or occasions, such as Aid-e-Ghorban (related to Hadj Pilgrim) in the Muslim countries such as Iran, weddings, birth of first child, especially birth of a boy or other traditions, which involve the ritual home-slaughter of thousands of sheep, cattle and even camel, could be a source of dogs’ infection. This practice favors the spreading of the parasite in owned and farm dogs and contributes to maintaining high infection rates in these dogs and in ruminants and humans (21–23).

Several recent provinces–wide and countrywide surveys of hospital records have conducted and reported thousands of surgical human cases different genotypes of the parasite (24, 25). Based on review of the origin of hospital cases, the majority of reports classified the most parts of country as hyper endemic zone (surgical incidence > 5 cases per 100000 populations). Hyper endemic zone includes the pastoral and agricultural regions in the slop of Zagros and Alborz mountains. In some areas, stray dog infection rate to *E. granulosus* reported higher than 50%, even 60% (26, 27).

As for age in this disease, in Iran, most patients cases with symptomatic disease were between the ages of 2.5 through 85 yr. Rates were similar in both sexes (7). In that report, most cases were in adults aged 21–40 yr and infection rate was relatively higher in females.

Unfortunately, systematically collected hospital based data are not available from all countrywide hospitals, even public or university hospitals. Therefore, the health system of country lack of the comprehensive and precious information on the prevalence and incidence of human or even animal echinococcosis, so local surveys could be useful for estimation of problem burden at least at the regional level.

Control of parasite by solely administering anti-helminthes to dogs, or occasionally stray dogs destroy by gun or toxic bait is not too effective in developing countries where the disease is endemic. Determining main hosts in each community by molecular methods, that playing main role in the epidemiology of disease, especially main source of human infection, could provide useful information for control strategy (28, 29). Advances in knowledge and development/design of new control tools for hydatid disease including new diagnostics and antiparasite vaccines for the definitive and intermediate hosts provide an excellent prospect for improved control programs.

## Conclusion

This study indicates human hydatidosis is still a major public health problem in this province, in spite of significant decrease in the prevalence of other parasitic infections. This disease was burdening huge treatment and economic costs to people in this province.
